# Combining Photogrammetry and Photometric Stereo to Achieve Precise and Complete 3D Reconstruction

**DOI:** 10.3390/s22218172

**Published:** 2022-10-25

**Authors:** Ali Karami, Fabio Menna, Fabio Remondino

**Affiliations:** 1 3D Optical Metrology (3DOM) Unit, Bruno Kessler Foundation (FBK), 38123 Trento, Italy; 2 Department of Information Engineering and Computer Science, University of Trento, 38123 Trento, Italy

**Keywords:** photogrammetry, photometric stereo, high-resolution 3D reconstruction, point cloud, non-collaborative objects, industrial metrology

## Abstract

Image-based 3D reconstruction has been employed in industrial metrology for micro-measurements and quality control purposes. However, generating a highly-detailed and reliable 3D reconstruction of non-collaborative surfaces is still an open issue. In this paper, a method for generating an accurate 3D reconstruction of non-collaborative surfaces through a combination of photogrammetry and photometric stereo is presented. On one side, the geometric information derived with photogrammetry is used in areas where its 3D measurements are reliable. On the other hand, the high spatial resolution capability of photometric stereo is exploited to acquire a finely detailed topography of the surface. Finally, three different approaches are proposed to fuse both geometric information and high frequency details. The proposed method is tested on six different non-collaborative objects with different surface characteristics. To evaluate the accuracy of the proposed method, a comprehensive cloud-to-cloud comparison between reference data and 3D points derived from the proposed fusion methods is provided. The experiments demonstrated that, despite correcting global deformation up to an average RMSE of less than 0.1 mm, the proposed method recovers the surface topography at the same high resolution as the photometric stereo.

## 1. Introduction

Image-based 3D reconstruction methods (in particular photogrammetry and photometric stereo) have been employed for a long time for reconstructing the 3D shape of objects, being cost-effective [1], portable [2] and flexible [3]. These methods are commonly used in various applications [4,5,6]. In particular, in the field of industrial metrology, they can be used for quality control [7,8,9,10], inspection [11,12,13,14], reverse engineering [10,15,16,17] or 3D micro-measurement [18,19,20].

**Figure 1:**
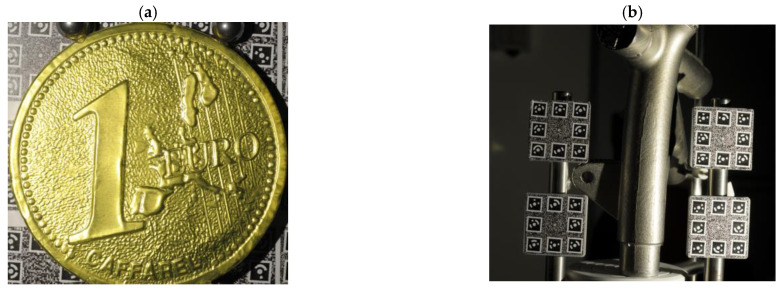
Examples of objects considered in this work and featuring non-collaborative surfaces (shiny, textureless). (**a**) a gold foiled surface shaped like a Euro coin featuring high reflective, very detailed structures [25]; (**b**) a metallic object with less reflectivity while featuring a geometrically complex shape [11].

**Figure 2:**
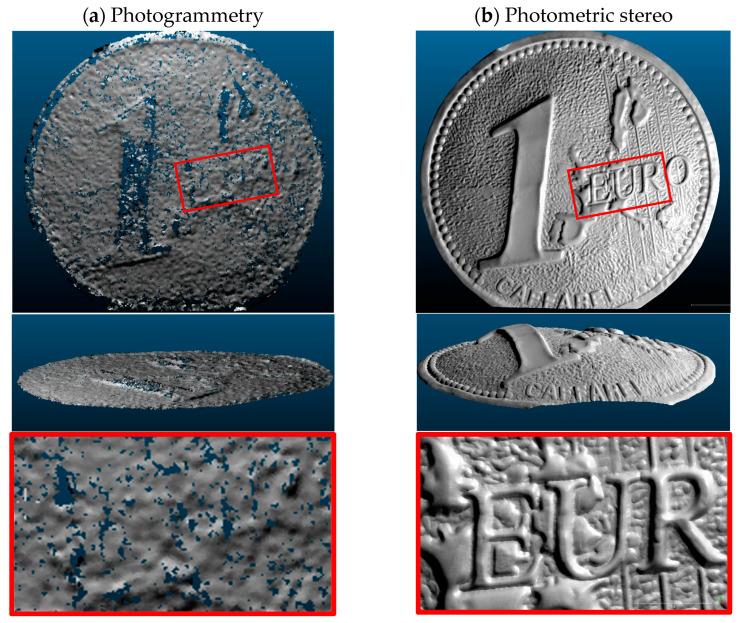
Visual comparison between photogrammetry and photometric stereo in terms of low and high-frequency information retrieved by the two techniques using the same dataset with the same configuration adopted from [25]. (**a**) accurate low-frequency information but noisy 3D details derived with photogrammetry; (**b**) high-details but deformed global shape derived with photometric stereo. Experiment details for this object (object F) with a comprehensive quantitative comparison are described in Section 5.

Photogrammetry can generate a geometrically accurate and dense model of a real-world object from a series of images of an object or a scene taken from various viewpoints under the assumption of known materials and lighting conditions [17,21]. However, it is still challenging to achieve high-accuracy 3D measurement of non-collaborative objects (Figure 1) due to the sensitivity of photogrammetry to the textural properties of the surface [22,23,24]. For example, when the surface of an object is featureless or displays repetitive patterns, all methods based on feature extraction face difficulties in finding a sufficient number of corresponding image points that are needed for image orientation [1,25]. In case of polished and shiny surfaces, such as industrial and metallic components (Figure 1), the incoming light is subject to the law of reflection and is observed as specular reflection. Such reflections, present in captured images, are undesirable and dense image matching procedures produce noisy results on high-reflective and poorly textured objects (see Figure 2a).

Photometric stereo, on the other hand, is an effective method able to retrieve surface normal using a set of images captured under various lighting conditions [26] and applying the gradient field [27,28,29] to directly compute object depth from surface normals. This technique can recover a very detailed topography of objects even with texture-less or shiny surfaces [29,30,31]. Indeed, as the photometric stereo technique requires images captured under multiple light directions, the problem of specular reflection is partially mitigated. However, a global deformation of the recovered 3D shape is generally present (Figure 2b) due to unfulfilled assumptions and to simplifications made to the mathematical model on how light interacts with the object surface [32,33], in particular:

1. The surface of the object should have an ideal diffuse reflection with no shadow and specularities on the surface.
2. Light rays arriving at the surface should be parallel to each other.
3. Camera uses an orthogonal projection.

Furthermore, 3D data generated using a photometric stereo are produced up to a scale factor, and accurate scaling is not as straightforward as other techniques—such as photogrammetry.

The presented work reports an integration methodology to successfully survey objects with non-collaborative surfaces (Figure 1). The approach integrates photogrammetry with photometric stereo in order to overcome their individual limits and reconstruct an accurate and high-resolution topography of non-collaborative surfaces. First, we use photogrammetric 3D measurements to provide scale and accurate low-frequency measurements where photometric stereo fails. Then, with a photometric stereo, we recover the high detailed topography of the object. Finally, we co-register and fuse all obtained 3D measurements to generate a precise and complete 3D reconstruction of non-collaborative objects. The paper builds upon [11,25], in which they only present the main concept and high-level architecture of the prototype system developed in the FBK-3DOM laboratory to synergistically combine photogrammetry and photometric stereo methods. They discuss one of its main components, for instance, the concept of utilizing light directionality to increase texture quality by exploiting shade and shadow phenomena in detail and only addressed parallel light direction without taking intensity attenuation into account. However, this article provides more details in the following sections, particularly for the data integration approach with more critical and comprehensive analyses. Furthermore, this article not only addresses angular and radial intensity attenuation but also detects and removes shadow and specular reflection. Additionally, as a final correction, we propose three different global/local shape correlations to mitigate shape deformation. In summary, the main contributions are as follows:

1. A semi-automatic image acquisition system based on the near-field photometric stereo lighting system and suitable for integrating photogrammetry measurements and photometric stereo;
2. An algorithm for removing specular reflection and shadow, as well as determining lighting direction and illumination attenuation at each surface point, using the accurate geometry of the lighting system and the object’s sparse 3D shape.
3. Three different approaches to take advantage of photogrammetric 3D measurements to correct the global shape deviation of photometric stereo depth caused by violating assumptions such as orthogonal projection, perfect diffuse reflection, or unknown error resources.

The rest of the paper is organized as follows: Section 2 reviews the related investigations and uses of photogrammetry, photometric stereo and their combinations. The proposed fusion methodology is introduced in Section 3. Section 4 presents the proposed framework for data acquisition, along with its calibration steps. Section 5 reports 3D reconstruction of non-collaborative surfaces and essential analyses using the proposed algorithms. Finally, conclusions are drawn and presented together with future research plans.

## 2. State of the Art

### 2.1. Photogrammetric Methods

Photogrammetry has historically and widely been regarded as one of the most effective techniques for 3D modeling of well-textured objects. Photogrammetry allows for recovering the 3D shape of the object accurately and reliably compared to photometric stereo. However, regions with poorly texture or repetitive patterns are difficult to reconstruct since all reconstruction methods of this kind require matching correspondences in various images [22,23]. Over the years, various photogrammetric methods have been developed to deal with the 3D reconstruction of such non-collaborative objects. In the case of textureless Lambertian objects, several solutions for enhancing the surface texture are suggested with, for example, the projection of known patterns [3,34], random [1,2], or synthetic [23,24] ones onto the object surface. For example, Ahmadabadian et al. [2] established a relatively inexpensive automated image acquisition system used for 3D modeling of textureless objects that work by projecting a noisy pattern onto the examined object. Menna et al. [3] have developed a similar automatic workflow based on the known pattern projection such as a structured-light pattern for 3D digitization of heritage artifacts. Methods based on the pattern projection improve the surface texture and, as a result, the accuracy of the final 3D reconstruction when dealing with only Lambertian surfaces [34]. However, these methods have problems when dealing with highly reflective surfaces with heavy specular reflection or interreflection [2,34]. In the case of reflective objects, cross polarisation [35,36] and image pre-processing approaches [37,38,39] have also been employed to decrease specular reflections. However, these procedures may smooth off surface roughness or vary the texture from one view to the next, negatively affecting the results [11]. Another common approach to spray the surface with a thin layer of white or colored powder [1,2,25,40,41,42] can also be used as a common solution. However, powdering the object surface might be impractical when the real topography of an object is needed at high-resolution since the layer added increases the total object volume and can smooth out minor information. In addition, surface treatment is impossible in the case of delicate cultural heritage assets or real-time 3D surface inspection [17,41,42].

### 2.2. Photometric Stereo

Photometric stereo is a technique for estimating an object’s surface normal using the illumination changes, which was first proposed by Woodham [26]. Over the years, many techniques [28,29,43] have been developed to extract the geometry of objects from surface normals using a gradient field. However, the classical photometric stereo approaches work with perfectly diffuse (Lambertian) surfaces, which is often an improper assumption for many objects such as metallic, glossy, and shiny. Therefore, the performance of such techniques degrades on real-world objects, which frequently exhibit non-Lambertian reflectance such as interreflection and specular reflection [4,29,32]. To address these issues, different approaches have been developed over the years. The first group of approaches classify and remove the specular highlights when dealing with non-Lambertian surfaces. For example, earlier approaches [44,45] used three illumination directions out of four at each surface point in which the surface seems more Lambertian to approximate the direction of the surface normal. Following this, several algorithms were proposed based on RANSAC [46,47], the median values [48,49], graph cuts [50], maximum-likelihood estimation [50,51], using robust SVD [52], or Markov random field [53] to extract Lambertian images in a more stable form. However, more input images are also needed for statistical analysis. Moreover, their output negatively affects complex objects with features such large interreflection and specular reflection since the non-Lambertian outliers are dense [32,54].

Instead of discarding specular reflection as outliers, the second group of investigations fitted a nonlinear analytic Bidirectional Reflectance Distribution Function (BRDF) to model the behavior of the light. In this regard, different BRDF models have been developed such as the Ward model [55], the Torrance–Sparrow model [56,57], the specular spike [58], bivariate BRDF [59,60], symmetry-based approach [61], spatially-varying BRDF [62], etc. Unlike the previous group, they have the benefit of using all available data. The downside to such methods is that analytical models vary considerably from one object to the next, and each is confined to a specific material class. Such approaches also require a complex case-by-case analysis of different content classes in theory [32,54].

Photometric stereo-based methods, unlike photogrammetry techniques, can reconstruct a very detailed surface’s topography even with non-collaborative objects [63,64,65]. However, owing to some mathematical assumptions, such as parallel light direction and orthogonal projection of the sensor, the global shape deformation typically exists [4,29,32,66]. The global shape deviation can vary depending on the surface properties and dimensions of the object. For instance, the generated 3D reconstruction can be deformed with a maximum shape deviation of about 13 mm on a Lambertian flat object with 340 × 270 mm dimensions when ignoring the assumptions mentioned above [25,66].

### 2.3. Combined Methods

Various researchers combined photometric stereo with other techniques such as structured light or photogrammetry. In the developed methods, high-frequency spatial information is recovered from photometric stereo, whereas other techniques are applied to retrieve low-frequency information. For example, Smithwick and Seibel [67] proposed a Single Fiber Scanning Endoscope (SFSE) system for generating dense range maps and 3D measurements based on the merging of photogrammetric disparity and photometric stereo methods, providing precise volume measurements for dosage, risk estimate, and healing progress analyses. Nehab et al. [68] combined 3D reconstruction generated from a range scanner with photometric normals to improve the accuracy and level of detail. Hernandez et al. [69] used a multi-view geometric constraint from shape from silhouette (SFS) to mitigate photometric stereo’s low-frequency surface distortion. Although this method is simple and flexible, it works only with particular parametric BRDF models [70]. Several works [51,71,72] combined photometric stereo with RGB-D sensors to derive the 3D details from photometric stereo while improving the low frequency information using RGB-D data. Later, Park et al. [73,74] then suggested an uncalibrated multi-view photometric stereo (MVPS) approach for recovering precise 3D reconstruction of the object utilizing a coarse mesh with a 2D displacement map. However, the approach is unable to reconstruct objects with a wide range of surface reflectance characteristics as well as textureless surfaces [29]. Logothetis et al. [75] proposed a new MVPS approach capable of modeling objects with complex geometry where occlusions and/ or cast shadows may have occurred. More recently, Ren et al. [4,76] integrated a photometric stereo with sparse 3D points generated using contact measurements (CMM) to correct the global distortion caused by a photometric stereo. The use of expensive technology restricts the method to special laboratories and projects with particular metrological demands, despite the fact that these systems may achieve high precision performances. Li et al. [29] developed an MVPS approach in which a sparse 3D point is used to improve the geometry of the depth map generated by a photometric stereo. However, this procedure includes explicit geometric modeling stages such as multi-view depth propagation, iso-depth contour estimation, and/or tracing contours, which must be processed and completed properly in order to obtain a 3D reconstruction of the surface, making it more difficult, time-consuming, and challenging. Furthermore, they used a turntable to rotate the object while keeping the camera and light sources fixed in order to capture multi-view images, which means that the light sources are not constant from one view to the other. This could change the object texture from one view to the other resulting in noise or false matching during the image orientation and dense matching process. A few works have recently investigated using different learning-based approaches [70,77] to fuse photometric stereo and MVS for effectively utilizing their complementary strengths. Although these approaches are simple and easy to use, they are much less precise than traditional integration methods, making them unsuited for industrial applications where 3D measurement precision and reliability are required. Furthermore, training such algorithms necessitates large datasets labeled for a unique object type, making generalization to real-world objects problematic.

## 3. Methodology

**Figure 3:**
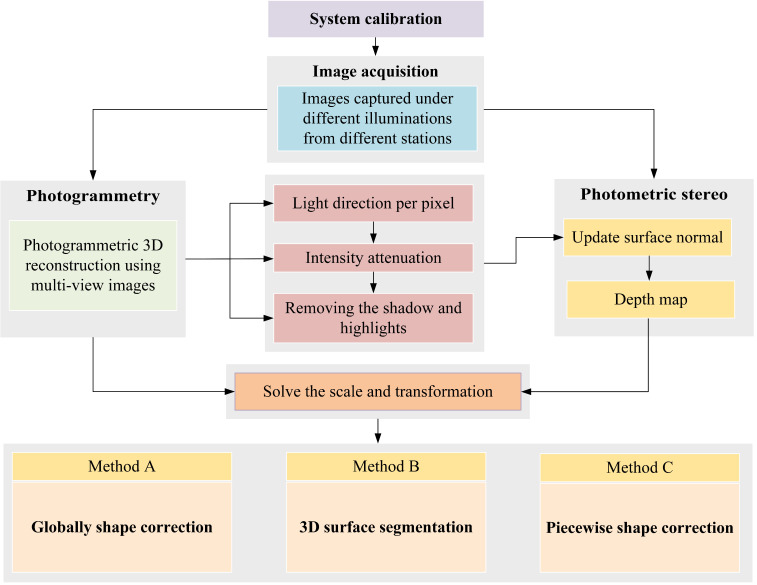
The general overview of the proposed integration with its three methods.

This research proposes a method for the 3D reconstruction of non-collaborative surfaces which combines photogrammetry and photometric stereo taking advantage of both methods and overcome their own limits. The proposed method is summarized in Figure 3. The first step is to provide an automatic image acquisition system to capture images under different illuminations and from different camera stations (camera positions) to satisfy the input requirements of the integrated method. A 3D point cloud is then generated with a photogrammetry pipeline [11]. The photogrammetric 3D shape measurements and the calibrated light positions are utilized to compute light direction and intensity attenuation (radial and angular) at each surface point. The initial surface normal is then recovered given the light direction and the corresponding intensities at each surface point. Following this, the object’s regions with shadow and specular reflection are detected and removed on the captured images depending on the angle between the light direction and the initial surface normal at each surface point. After removing outliers from images, the surface normal is updated given the light direction and the corresponding intensities at each surface point. A depth map is afterward generated from the integration of the surface normal. 

The scale factor is computed using corresponding 3D points between photogrammetric 3D reconstruction and photometric stereo depth maps. At the final stage, to further mitigate the shape deformation errors, three different approaches are proposed as follows:

1. **Method A**: it corrects the shape deviation by applying polynomial adjustment globally on the whole object;
2. **Method B**: it segments the object based on the normal and curvature and then applies the shape correction procedure on each segment separately;
3. **Method C**: it splits the object into small patches and then applies the shape correction procedure on each patch separately.

### 3.1. Basic Photometric Stereo

Photometric stereo is a method to recover the surface normal from multiple images that are taken under different lighting directions. The mathematical form of photometric stereo [26,32], for each surface point (X), is expressed in Equation (1):

(1) $$I_{i}\left(X\right)=l_{i}\left(k\hat{n}\right)\;\;\left(i=1,2,\ldots ,t\right)$$

where $I_{i}$ is the intensity observed in the i-th image, $l_{i}$ is the normalized light direction of the i-th source, k is the Lambertain reflection albedo, and $\hat{n}$ is the normalized surface normal at each surface point (*X*), which is unknown.

If t>3, l can be inversed as $l^{-1}I=k\hat{n}$. However, when the vector of light direction (l) is non-square, the generalization of the inverse is computed by multiplying both sides of the Equation (1) with $l^{T}$ as follows:

(2) $$l^{T}I=l^{T}k\hat{n}\;{\left(l^{T}l\right)}^{-1}l^{T}I=k\hat{n}$$

If we consider $k\hat{n}$ as a vector, the length of this vector is k, and $\hat{n}$ is the normalized direction of that vector. Therefore, the surface normal and albedo can be recovered using Equation (3):

(3) $$k=∥k\hat{n}∥\;\hat{n}=\frac{k\hat{n}}{k}$$

In theory, at least three lighting directions (images that are taken under different illuminations) are required to recover the normal at each point. However, practically, more than three images are used to minimize noises involved in the process.

After computing the surface normal, the surface from gradients technique [27,32] is applied to generate a 3D shape of the object from the field of normal vectors. The depth map is specifically given as z=f(p, q), and the normal of the surface points towards the gradient direction, where the *p* and *q* values are obtained as $p=-\frac{n_{x}}{n_{z}}$ and $q=-\frac{n_{y}}{n_{z}}$.

### 3.2. Light Direction per Pixel

Conventional photometric stereo assumes that the light rays coming from the source are parallel. Providing parallel illumination conditions is more complicated and inefficient to implement in a close-range lighting system. Furthermore, it is also clear that rays coming from the light sources are not parallel anymore, especially when the lighting system is close to the object. Therefore, this impact must be addressed for accurate measurement of the normal surface as any change in light position negatively affects the normal and consequently the resulting 3D reconstruction. For these reasons, we explored a geometric model with punctiform light sources and light divergence as expressed in [11,25].

To compute a unique light direction at each surface point, the 3D shape of the object and the light positions must be known in the same reference system (camera coordinate system). For doing this, 3D points ($P_{s}(X,Y,Z)$) and the light positions ($l_{k}$) are transferred to the camera coordinate system using exterior orientation parameters [11,25,78]. Then, the sparse 3D points are then back-projected to the camera coordinate system using the collinearity equation and known interior orientation parameters to find their corresponding pixels on the image ((*I*(*u*,*v*))). Then, as expressed in Equation (4), the normalized light direction ($\hat{v_{k,s}}$) for each surface point ($P_{s}\left(X,Y,Z\right)$ is computed given the coordinate of the *k*th light source ($l_{k}$) [25,66]:

(4) $$\hat{v_{k,s}}=\frac{\left(l_{k}-p_{s\left(X,Y,Z\right)}\right)}{\left\|l_{k}-p_{s\left(X,Y,Z\right)}\right\|}$$

Finally, the surface normal is computed at each surface point given $\hat{v_{k,s}},$ and their associated image intensities (*I*(*u*,*v*)) as expressed in Equation (5):

(5) $$\hat{n_{s}}=\frac{{\left(v_{k,s}{}^{T}\cdot v_{k,s}\right)}^{-1}\cdot v_{k,s}{}^{T}\cdot I_{k}}{\left\|{\left(v_{k,s}{}^{T}\cdot v_{k,s}\right)}^{-1}\cdot v_{k,s}{}^{T}\cdot I_{k}\right\|}$$

### 3.3. Backprojection

As we explained in the previous section, in order to compute a unique light direction for each surface point, we need to have the 3D coordinate of each light source as well as the surface point in the camera coordinate system where the depth from the photometric stereo is computed. The 3D points and the light positions, measured during the calibration process, are transformed into the camera coordinate system using Equation (6):

(6) $$\left[\begin{array}{c} x\\ y\\ z \end{array}\right]=R\left[\begin{array}{c} X\\ Y\\ Z \end{array}\right]+t$$

where (*X*, *Y*, *Z*) are the 3D coordinates of a surface point or light source in the local coordinate system defined during the calibration step, and R and t are the rotation matrix and translation vector, respectively.

Successively, the 3D points are back-projected in the image coordinate system using the perspective transformation expressed in Equation (7), and then to the pixel coordinate system:

(7) [x′y′]=[xzyz]

Generally, the lenses used in any project have some distortion, which could be modeled as radial distortion and tangential distortion [79]. These distortions are modeled using Equation (8):

(8) $$\left[\begin{array}{c} x^{\prime \prime }\\ y^{\prime \prime } \end{array}\right]=\left[\begin{array}{c} x^{\prime }(1+k_{1}r^{2}+k_{2}r^{4}+k_{3}r^{6}+k_{4}r^{8})+(p_{1}(r^{2}+2{x^{\prime }}^{2})+2p_{2}x^{\prime }y^{\prime })(1+p_{3}r^{2}+p_{4}r^{4})\\ y^{\prime }(1+k_{1}r^{2}+k_{2}r^{4}+k_{3}r^{6}+k_{4}r^{8})+(p_{2}(r^{2}+2{y^{\prime }}^{2})+2p_{1}x^{\prime }y^{\prime })(1+p_{3}r^{2}+p_{4}r^{4}) \end{array}\right]$$

where:

$r^{2}={x^{\prime }}^{2}+{y^{\prime }}^{2}$;
$k_{1}$, $k_{2}$, $k_{3}$, and $k_{4}$ are radial distortion coefficients;
$p_{1}$, $p_{2}$, $p_{3}$, $p_{4}$ are tangential distortion coefficients.

After estimating the lens’s distortion, we can ultimately reach the pixel coordinate system by utilizing Equation (9):

(9) $$\left[\begin{array}{c} u\\ v \end{array}\right]=\left[\begin{array}{c} f_{x}x^{\prime \prime }+c_{x}+w0.5+B_{1}x^{\prime \prime }+B_{2}y^{\prime \prime }\\ f_{y}y^{\prime \prime }+c_{y}+h0.5 \end{array}\right]$$

where (u, v) is the coordinate of the image point in pixels corresponding to the reconstructed 3D points ($P_{s}(X,Y,Z$)), $f_{x}$, and $f_{y}$ are the focal lengths in pixel unit, and ($c_{x}$, $c_{y}$) is the coordinate of the principal point. $B_{1}$, $B_{2}$ are affinity and non-orthogonality (skew) coefficients, respectively; w, h are image width and height in pixels.

### 3.4. Intensity Attenuation

**Figure 4:**
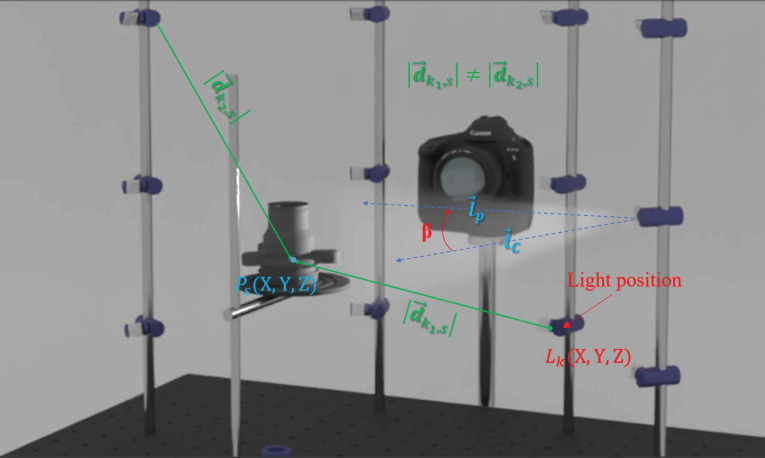
Radial and angular intensity attenuations.

There are two different kinds of light attenuation that are needed to be taken into consideration when point light sources are used (see Figure 4). The first factor is caused by a decrease in light energy that is proportional to the inverse squared distance between the light source to the surface point (radial intensity attenuation). The second attenuation factor that we address is a realistic directional model of a light source (angular intensity attenuation).

#### 3.4.1. Radial Intensity Attenuation

The intensity of the light decreases when the object moves away from the illumination source [80]. In theory, this behavior of the light is modeled using Equation (10):

(10) $$F_{k,s}^{R}=\frac{1}{h_{c}+h_{l}\left|{\vec{d}}_{k,s}\right|+h_{q}(\left|{\vec{d}}_{k,s}\right|{)}^{2}}$$

where $\left|{\vec{d}}_{k,s}\right|$ is the distance between the k-th light source and surface point ($P_{s}(X,Y,Z$)). $h_{c}$, $h_{l}$, and $h_{q}$ are the attenuation coefficients. However, in practice, the first and the second terms are ignored since their values are negligible compared to the third term. In this paper, we also considered $h_{q}$ equal to one.

#### 3.4.2. Angular Intensity Attenuation

The light intensity decreases as we move away from a light source, but also when the light moves angularly (*β*) further from the cone axis (${\vec{l}}_{C}$) [80]. Equation (11) is a commonly-used approach to model such a phenomenon:

(11) $$\cos{\left(\beta \right)}^{\mu }={\left({\vec{l}}_{C}\cdot {\vec{l}}_{s}\right)}^{\mu }$$

where *β* is the angle between the cone axis (${\vec{l}}_{C})$ and the direction from the light position to the surface point (${\vec{l}}_{s}$), and μ is the attenuation coefficient.

### 3.5. Shadow and Specular Reflection Removal

**Figure 5:**
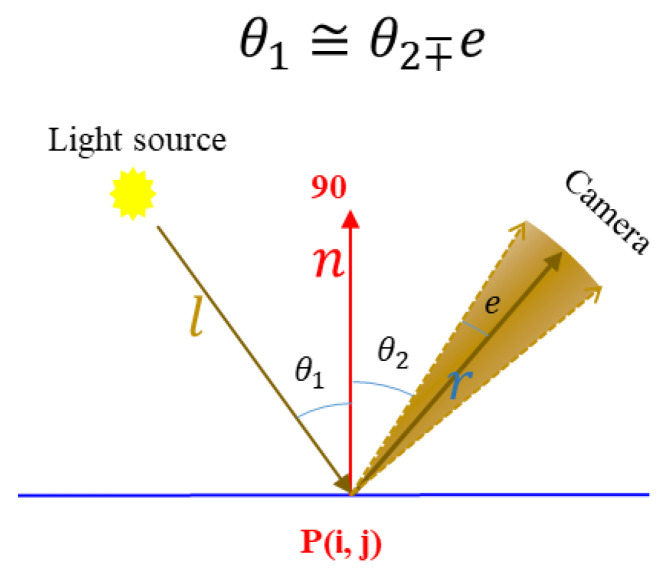
Removing shadow and specular reflection using the accurate geometry of the lighting system and object’s sparse 3D shape.

The known camera geometry, lighting system and approximate 3D shape of the object were used to automatically detect shadow and specular reflection and keep only the best-highlighted parts on each image where 3D microstructures and roughness are seen. After capturing multiple images from different stations, the images are automatically inspected, and those regions on each image that appear to result in inferior quality and/or noise (shadow and secularity) are excluded [11]. To do this, as shown in Figure 5, the incoming angle ($\theta_{1}$) and reflected angle ($\theta_{2}$) are estimated at each surface point *P*(*x*,*y*,*z*) corresponding to each pixel (*i*,*j*) given the light direction $l_{\left(x,y,z\right)}$, camera direction $r_{\left(x,y,z\right)}$ and normal $n_{\left(x,y,z\right)}$ using Equations (12) and (13):

(12) $$\theta_{1}=\cos^{-1}\left(\frac{n_{\left(x,y,z\right)}\cdot l_{\left(x,y,z\right)}}{∥n∥∥l∥}\right)$$

(13) $$\theta_{2}=\cos^{-1}\left(\frac{n_{\left(x,y,z\right)}\cdot r_{\left(x,y,z\right)}}{∥n∥∥r∥}\right)$$

Specular reflection occurs when an incoming light ray ($l_{\left(x,y,z\right)}$) reflects off of a surface point $P_{\left(x,y,z\right)}$ at an equal but opposite angle ($\theta_{2}$) to its incoming angle $\theta_{1}$. In our experiment, we consider a small amount of light scattering (*e*) around the reflection vector (*r*) as the specular reflection zone, which is tuned to ±2 degrees. This value can be changed depending on the strength of the light source. By increasing this value, we assure that a larger area around reflection vector (*r*) is considered as an outlier.

A self-shadowed pixel is shaded by itself. Geometrically, the angle between the surface normal (*n*) and the light source direction (*l*) is more than 90 degrees ($\theta_{1}>90$). In this experiment, a pixel is considered as a self-shadow if the computed incoming angle $\theta_{1}$ is larger than 85 degrees. Once self-shadow and specular reflection are removed from images, the surface normal is updated for further processing.

### 3.6. Helmert Transformation

The Helmert 3D transformation is one of the most often used transformation methods in geodetic applications. This transformation is defined with seven parameters, including three translations, three rotations, and a scale factor, which allow us to transform the photometric stereo depth map to a defined coordinate system from which photogrammetric 3D points are obtained. When this transformation is performed on a 3D point cloud, it rotates, transforms, and scales the point cloud with respect to the defined coordinate systems. The mathematical form of this transformation is expressed in Equation (14):

(14) $$X_{T}=C+\mu RX$$

where $X_{T}$ is the original 3D Points (3D reconstruction from photogrammetry), and *X* is the transformed 3D points (3D reconstruction from photometric stereo), *C* is the three translations ($x_{t},\;y_{t},z_{t}$) along the coordinate axes, *R* is the rotation matrix, and μ is the scale factor. The seven Helmert transformation parameters must be calculated using corresponding points from both datasets.

### 3.7. Global Shape Correction with Polynomial Model (Method A)

The 3D reconstruction computed using the photometric stereo principle shows residual deformation even after correcting the light directions and applying intensity attenuations because of the other mathematical simplifications and unknown error sources. Therefore, to further mitigate the residual errors, we use a polynomial mapping from the 3D model obtained with the photometric stereo to the one obtained through photogrammetry. This technique was inspired by polynomial adjustment in aerial triangulation [81] with 20 coefficients. The 20 coefficients are computed using least square principles after forming the design matrix containing the Equation (15)—one for each common point in the photogrammetric (*X*, *Y*, *Z*) and photometric stereo (*x*, *y*, *z*) models:

(15) $$X=x+a_{1}+a_{3}x-a_{4}y+a_{5}\left(x^{2}-y^{2}\right)2a_{6}xy+a_{7}\left(x^{3}-3xy^{2}\right)-a_{8}\left(3x^{2}y-y^{3}\right)\;Y=y+a_{2}+a_{4}x+a_{3}y+a_{6}\left(x^{2}-y^{2}\right)+2a_{5}xy+a_{7}\left(3x^{2}y-y^{3}\right)+a_{8}\left(x^{3}-3x^{2}y\right)\;Z=z+b_{0}-2b_{2}x+2b_{1}y+c_{1}x^{2}+c_{2}x^{3}+c_{3}x^{4}+d_{1}xy+d_{2}x^{2}y+d_{3}x^{3}y+d_{4}x^{4}y+e_{1}y^{2}+e_{2}xy^{2}$$

It is worth noting that both the deformed model (from photometric stereo depth map) and reference data (photogrammetric 3D points) should be approximately scaled and aligned before computing the coefficients. The disadvantage of such polynomial adjustment is that the edges and boundaries of complex-geometry surface objects can be smoothed out (e.g., Objects C and D). To preserve the edges for complex-geometry objects, two following approaches (Methods B and C) are afterward presented.

### 3.8. D Surface Segmentation (Method B) 

**Figure 6:**
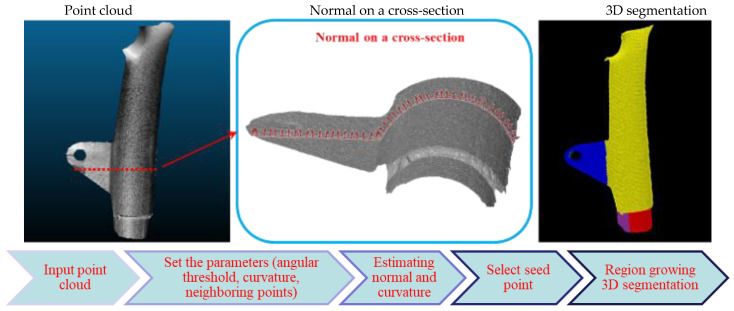
Schematic view of the region growing 3D segmentation approach (Method B). Each color represents a different segment.

A 3D segmentation is performed on the point cloud in order to divide the object into many parts, and then transformation and shape correction are applied to each segment separately (Figure 6). 

To segment the point cloud, a region growing approach [82] is adopted. This method uses local features (point cloud normal and curvature values) obtained from neighbouring points to segment nearby points with similar properties. After obtaining the k-NN for a point p, the salient local 3D features (e.g., point normal, curvature, etc.) are calculated for each point p. The point p with the minimum curvature value is chosen as the first seed point to begin the region growing process. Therefore, the point might be selected in a smoother area on the object where the surface variation is lower. Following the selection of seed points, the region growing segmentation starts and gradually expands by adding new points. Once a first segment is complete according to a region growing criteria [82], a new seed point is selected for the following segment.

### 3.9. Piecewise Shape Correction (Method C)

**Figure 7:**
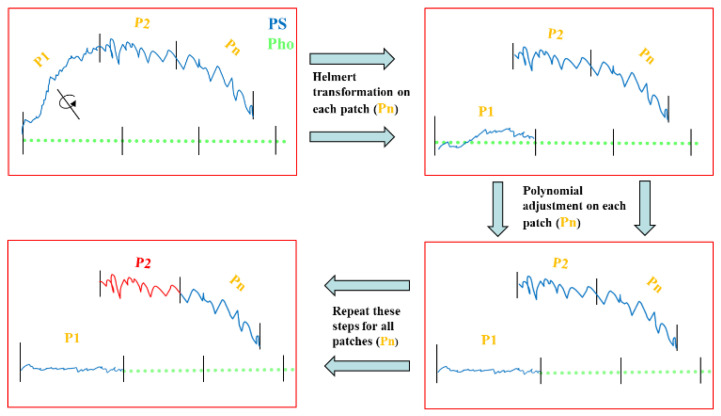
Schematic view of the piecewise shape correction approach (Method C).

The object is divided into small patches (Pn) as illustrated in Figure 7, and then the shape correction is applied to each patch (Pn) individually. As a result, the global geometry of the surface in each patch becomes less complicated, with fewer edges and boundaries; hence, the object’s deformation is more likely to be corrected more effectively. To this end, after splitting the object into small patches in the image space, we select the first patch and then, using the corresponding points in both models (photometric stereo and photogrammetry), the patch from the deformed model is roughly transformed and fitted to the course photogrammetric model using helmet transformation. Then, a polynomial adjustment is applied to the same patch to mitigate the deformation locally. This procedure should be performed for next patch ($P_{2}$) and the remaining patches. Although this approach corrects the global deformation, the model’s 3D details might be negatively affected near the patch’s borders due to disconnectivity. To solve this, two constraints are considered: (i) each patch must have an overlapping area with its neighbouring patches, and (ii) there must be always some corresponding points in the overlapping area to stitch all the patches together.

## 4. Data Acquisition System 

### 4.1. Imaging Setup

**Figure 8:**
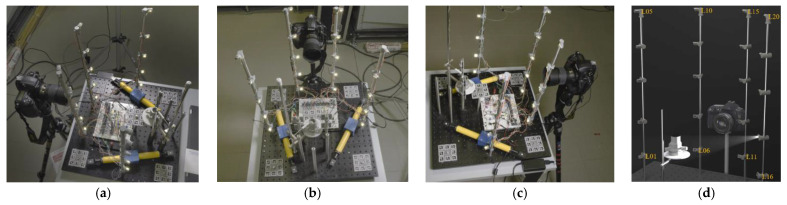
The proposed image acquisition system for combining photometric stereo and photogrammetry(**a**–**c**). The configuration of the overall lighting system (**d**).

The preliminary design of the proposed image acquisition workflow is presented in Figure 8. The system is composed of four main parts [11,25]: (1) a digital camera fixed at a distance from the object (400 mm approximately) depending on the needed GSD (Ground Sample Distance); (2) multiple dimmable LEDs lights on vertical poles (currently some 20 LEDs on four vertical poles); (3) a support to place the object to be surveyed; and (4) a microcontroller (Arduino) with electronic circuitry to manage the synchronization and control the camera and LEDs. 

A Nikon D3X DSLR camera with a resolution of 24 Mpx mounting two different lenses (AF-S VR Micro-Nikkor 105 mm f/2.8G, and AF-S Micro NIKKOR 60 mm f/2.8G ED) is used to capture the images. Once the object is placed on the rotating table, the camera is mounted on an adjustable-height tripod at the first station (about 400 mm from the object). The camera parameters, i.e., distance to the object, focal length, F-Stop, and ISO, are manually set by an operator and kept constant. To begin image acquisition, the following sequence is implemented: turn on the first LED, take an image, turn off the LED, turn on the second LED, and capture the second image. This procedure is carried out again for the remaining LEDs (twenty LEDs). Images are obtained within five seconds. Afterwards, the camera is moved to the next location, and the image acquisition process is repeated.

### 4.2. System Calibration

Before image acquisition starts, the system needs to be calibrated. Here, the goal is to establish the geometry of the lighting system and compute the camera interior and exterior parameters. A simple yet effective way is to use some coded targets embedded in the scene within the photogrammetry pipeline. The coded targets are arranged in a cluster of 8 on each plate, allowing for computing the 6 degrees of freedom (6DoF) of the target plate, useful to check its mechanical stability over time with respect to the other target plates. Targets are also placed vertically behind the inspected object and are measured with respect to the fixed reference system on the optical breadboard. In this way, whenever an image is captured, the camera pose can be registered to the fixed reference system of the optical breadboard. Two certified and accurately measured bars are first placed on the optical board to scale the reference coordinate system.

The light source coordinates are also computed during the calibration process. To do this, all LEDs are switched on and measured on the captured images as circular targets using the weighted centroid. The whole scene is captured from about 50 different stations, ensuring that a geometrically robust network of camera stations including convergent and rolled images is established. The images are oriented using standard photogrammetric procedures with a self-calibrating bundle adjustment to solve for camera’s internal and exterior parameters as well as 3D object points coordinates of coded targets and LED centers in a local reference coordinate system defined on the breadboard.

### 4.3. Testing Object

**Figure 9:**
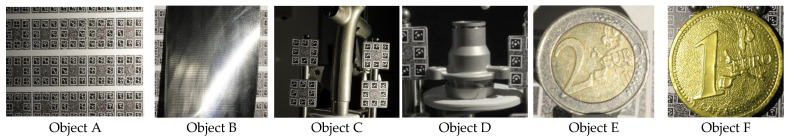
Used objects with their different surface characteristics.

Various objects (Figure 9) with complicated geometry, poor texture, metallic, and shiny surfaces are used for the assessment. Object A is a flat plane covered with a sheet of printed paper that provides a rough surface finishing. The paper shows a printed pattern of circular coded targets and a surrounding random texture. The object can be considered a good Lambertian approximation scattering the incident illumination in all directions equally. On this object, there are no self-shadows or specular highlights in the captured images. Object B is metallic and shiny with a curved shape. The phenomenon of interreflection makes it more complex compared to the other objects. Objects C and D are metallic with less reflectivity while featuring a geometrically complex shape. The image acquisition characteristics of these objects are reported in Table 1.

Object E is a two-euro coin, and object F is a gold foiled surface shaped like a Euro coin. The surface of objects E and F are very reflective, with very detailed structures. These objects are good examples to emphasize the proposed method’s capability for recovering microstructures on the surface while keeping the low-frequency information.

## 5. Experiments and Discussion

The proposed method (Figure 3) is evaluated on the six non-collaborative objects reported in Section 4. For each object, a set of images with GSD of ≈20 µm are acquired from three station using the proposed image acquisition system. From each station, multiple images are acquired under twenty different illuminations.

**Figure 10:**
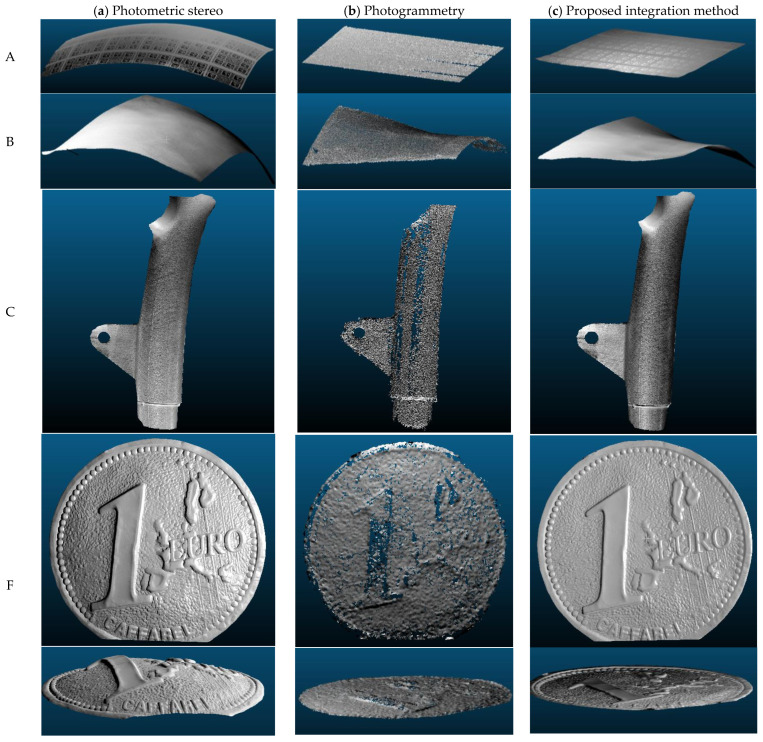
3D reconstruction generated using basic photometric stereo (**a**), photogrammetry (**b**), and proposed integration method (**c**) on four different non-collaborative objects (A–C and F).

A 3D point cloud with a photogrammetric pipeline is generated (refer to Karami et al. [11] for more details) using images taken from three different stations. The 3D coordinates of LEDs (as obtained during system calibration) and 3D object shape are then utilized to estimate light directions and intensity attenuations at each surface point. Regions with shadows and specular reflections are detected and masked out from the captured images, given the estimated light directions and the initial normal at each surface point. Following that, the surface normal is computed at each surface point given the light directions and intensities (only multiple images from first station are used to generate surface normal). The depth map is then generated from the integration of the surface normal. Using the interior and exterior orientation camera parameters, the estimated depth map is transformed to the same coordinate system where the photogrammetric 3D point cloud is reconstructed. The scale factor is computed using corresponding points between photogrammetric 3D reconstruction and the refined photometric stereo depth map. Finally, the three proposed methods A, B, and C described in Section 3 are used to adjust the remaining global deformation of the estimated photometric stereo depth map. Figure 10 presents some examples of the 3D results obtained using the proposed integrated method with respect to those achieved using a photometric stereo implemented by Xiong et al. [83] and photogrammetry.

The obtained 3D results indicate the clear advantage of the proposed integration. The proposed approach took the advantages of photogrammetry and photometric stereo to generate a reliable and high-detail 3D reconstruction of the non-collaborative objects. Indeed, thanks to the inclusion of photogrammetric 3D measurement, the global shape deviation, caused by assumptions and unknown error resources, is greatly mitigated (Figure 10c). Photogrammetric 3D reconstruction (Figure 10b) provides accurate geometric information compared to 3D photometric stereo (Figure 10a), where the generated 3D reconstruction is globally deformed. The proposed integrated algorithm reduced the global shape deformation aided by photogrammetry while keeping the 3D details from the photometric stereo.

### 5.1. Low Frequency Evaluation

In order to provide precise and reliable reference data for low frequency evaluation, a hexagon active scanner called AICON Primescan [84] with a nominal accuracy of 63 µm is used to scan the objects B, C, and D. In addition, an Evixscan 3D Fine Precision with a spatial resolution of 20 µm is used to scan object E. A geometric constraint, the best-fit plane, provides the reference for object A since the object can be considered to be planar. Since the object’s laser scanner 3D model is unavailable for object F, an additional photogrammetric 3D reconstruction is employed as reference data since its low-frequency information is still accurate. To generate this dataset, 30 additional images are taken [25]. Two different tests (cloud-to-cloud comparison and profiling) are accomplished using different objects.

#### 5.1.1. Cloud-to-Cloud Comparison

**Figure 11:**
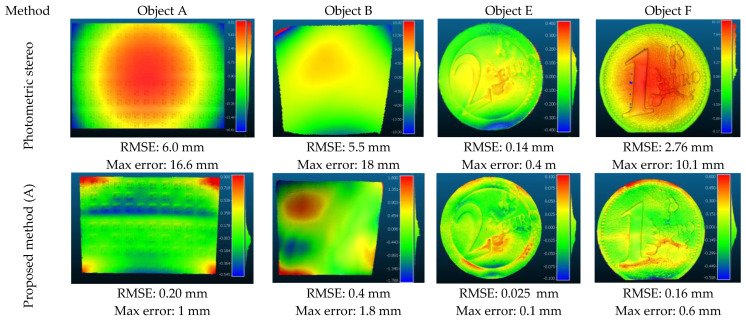
Cloud-to-cloud comparisons with reference data for basic photometric stereo and the proposed method (method A) on objects A, B, E, F.

To provide a cloud-to-cloud comparison, all the ground truth data were registered and transferred to a defined coordinate system (from which photogrammetric 3D points are obtained) using an Iterative Closest Point (ICP) technique [85]. The RMSE of the Euclidean cloud-to-cloud distances between the 3D points on the reconstructed and reference models is then computed and compared in CloudCompare. Figure 11 presents the cloud-to-cloud comparisons for the basic photometric stereo and the proposed approach (Method A) on four objects.

It can be seen that the highest low-frequency error belongs to object B with a RMSE of 0.4 mm. This is because the object’s size is quite large with a complex shape and a high reflecting surface, making the 3D reconstruction challenging.

However, there is a dramatic improvement compared to photometric stereo (RMSE of 5.5 mm). The low-frequency error for the rest of the objects is less than 0.2 mm proving that the proposed integration method can reduce the global shape deformation of 3D reconstructions.

**Figure 12:**
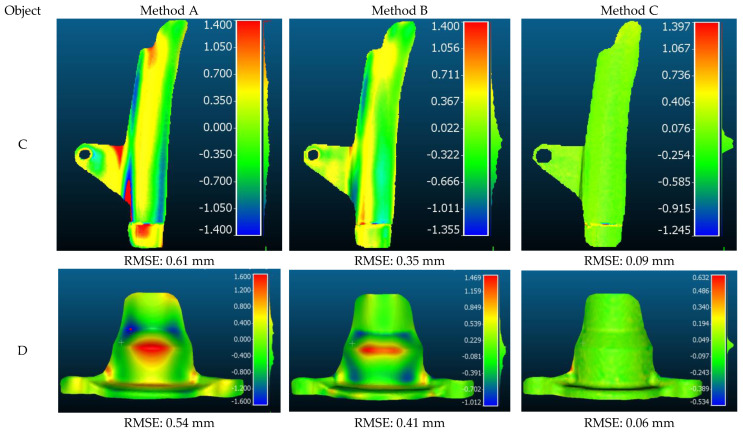
Cloud-to-cloud comparisons for the proposed methods on objects C and D featuring complex geometry.

It can also be shown that the proposed approach achieves larger errors in the boundaries (see Objects A and B—Figure 11). This is because there are no control points near the boundaries making it difficult to predict and modify correctly the 3D model outside of the control points using such a polynomial model. Furthermore, another downside of performing polynomial adjustment globally (Method A), as previously stated, is that the edges and boundaries of complex-geometry surface objects (e.g., Objects C and D) can be smoothed out. Therefore, to preserve the edges of objects with complicated geometry, methods B and C are proposed. The proposed methods are tested on two objects with complex geometry (C and D). The comparative 3D results after final shape correction using three proposed methods are presented in Figure 12.

As shown in Figure 12, the worst results for both objects are obtained by method A where the shape correction is applied globally at once on the object. This is because the surface of the objects is geometrically complex with more edges and borders; hence, applying such polynomial can smooth out the edges and boundaries, negatively affecting the low frequency information. However, these results are slightly improved for method B, where the object is first segmented and divided into small parts and then applied shape adjustment to each segment independently. For instance, the estimated RMSE in method A for object C is 0.61 mm while this value for method B decreased to 0.35 mm. The disadvantage of method B is that the 3D segmentation of the object takes time. Furthermore, the segmentation outcome is constantly dependent on certain input parameters, making precise segmentation of the object problematic. The final result is directly depending on segmentation part and therefore it is not always reliable.

On the other hand, method C significantly delivered the best results, with RMSEs of 0.09 mm and 0.06 mm for objects C and D, respectively. This is because the object on the image is split into small grid patches; then, piecewise shape correction is applied on each patch. Therefore, the global geometry of each patch became less complicated, almost flat with no edges and boundaries.

#### 5.1.2. Profiling

**Figure 13:**
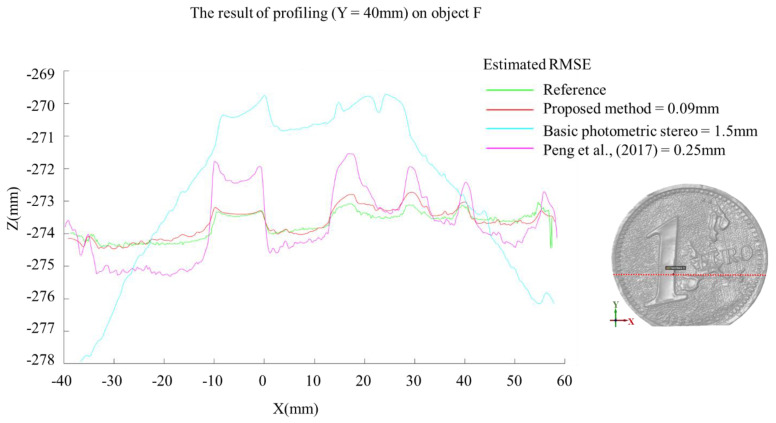
The comparison result of profiling for object F. The green profile represents the reference data (photogrammetry), the red profile represents proposed method, the blue represents basic photometric stereo, and the magenta profile represents the algorithm implemented in [51].

Profiling, or the extraction of a cross-sectional profile, is another helpful criterion to evaluate the performance of the proposed method. A cross-sectional profile can display the linear route of the obtained 3D points on a perpendicular plane which provides well-detailed geometric features of the profile. The object F is considered to be evaluated for this test. As shown in Figure 13 with different colors, four cross-sectional profiles are extracted and evaluated using photogrammetry, basic photometric stereo, the proposed method, and the algorithm implemented in [51]. The extracted section in each dataset is geometrically compared against the photogrammetric dataset using the well-known formula of Root Mean Square Error (RMSE). In Figure 13, the green line presents the photogrammetric cross-sectional profile while the red, blue, and magenta ones represent the basic photometric stereo, the proposed method, and Peng approach, respectively. From Figure 13, it can be seen that the proposed cross-sectional profile (red line) shows smaller errors (RMSE of 0.09 mm) and is closer to the photogrammetric section (green line) compared to the other approaches.

### 5.2. High Frequency Evaluation

**Figure 14:**
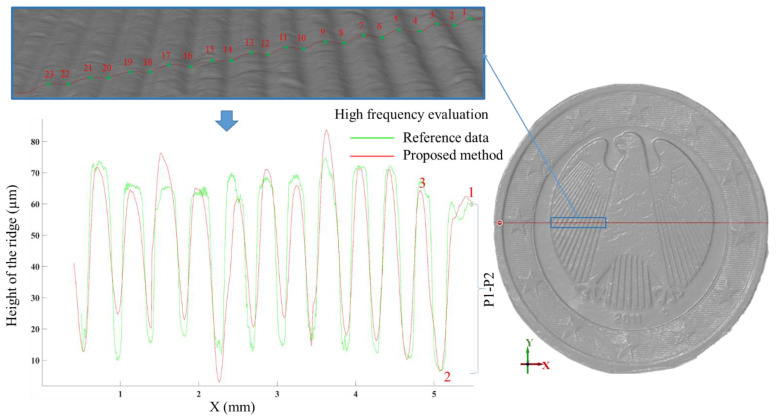
High resolution evaluation between the profiles measured with the profilometer in green and the proposed method in red for object E.

In order to evaluate the accuracy of the reconstructed high frequency information by the proposed methodology, the obtained 3D results are compared against reference data collected with a contact-type profilometer (Mitutoyo, Surftest SJ-210 [mm]; R2 µm; 0.75 mN; Item number: 178-560-11D). The profilometer has a diamond stylus of radius 2 µm and is used to measure a profile on the surface of object E to provide an accurate high-frequency profile. Then, the reference profile (the green profile in Figure 14) is compared to the same profile generated on the 3D data obtained by the proposed method (the red profile shown in Figure 14). The height of ridges on both extracted profiles is measured and compared. The results of this comparison are shown in Table 2, Table 3 and Table 4. From the achieved results, it can be seen that the estimated ridge heights for the proposed method is quite close to the ground truth provided by the profilometer. For instance, a maximum residual of 15.36 µm is measured for ridge P14-P15 while the estimated RMSE and Mean Absolute Error (MAE) for all ridges are about 1.5 µm and 5.48 µm, respectively.

The achieved results generate a highly-detailed 3D reconstruction of the surface topography, with a high level of agreement with the ground truth.

### 5.3. Comparing against State-of-the-Art

The proposed fusion methodology was compared against some state-of-the-art approaches in order to evaluate its performance. In particular, we considered only method A since it produces the poorest outcomes when compared to the other proposed approaches (B and C). The following methods from the literature were selected because they could potentially be applied to our datasets and be evaluated without the need for additional parameters or preparation:

Xiong et al. [83] combined local patch-wise inference with a global harmonization step for extracting a concise representation of the shape information available from diffuse shading in a small image patch.

Quéau et al. [50] presented a variational strategy for the robust photometric stereo method. This approach is explicitly introduced to deal with non-collaborative objects featuring self-shadows, cast shadows, and specularities. 

Peng et al. [51] utilized uncelebrated photometric stereo in combination with RGB-D sensors to extract high frequency information from photometric stereo while enhancing low frequency information with RGB-D data.

The 3D reconstruction results obtained by each algorithm are compared with the reference data and RMSEs of Euclidean cloud-to-cloud distances are calculated. The quantitative results of this comparison are shown in Table 5.

For each object, the blue values of Table 5 represent the best performance, while the red values represent the lowest performance. For all three objects, our proposed method achieves the lowest RMSE values, with the best result for object E (0.025 mm). For a complicated case, such as object D, which include shadows and interreflections, the estimated RMSE for the proposed method is 0.54 mm while this value for the other approaches is above 1.1 mm. This indicates the proposed method’s capacity to handle a variety of non-collaborative objects with self-shadows and high reflection, as well as complicated free-form surfaces, emphasizing its flexibility and robustness.

## 6. Conclusions and Future Works

The paper presented an approach, based on the combination of photogrammetry and photometric stereo, to take advantage of both methods for recovering surface microstructures while keeping the low-frequency information. Accurate geometric information like scale and low-frequency information is generated in areas where photogrammetric data are trusted. On the other hand, a highly detailed topography of the surface is recovered using photometric stereo which offers high-spatial resolution capacity. Three different methods are proposed to correct global shape deformation using 3D photogrammetric measurements. Six different objects with different surface characteristics and different comparative analyses are used in order to test and evaluate the proposed method. For each object, a set of images with GSD of ≈20 µm are acquired under twenty different illuminations from three stations using the proposed image acquisition system. Various tests, including cloud-to-cloud comparison and profiling, are performed using reference data to evaluate the proposed method in terms of low and high-frequency information. The proposed integrated approach recovered high-resolution details similarly to photometric stereo while inheriting the geometric information from photogrammetry with the RMSE of less than 0.1 mm.

In future works, we will also employ more synchronized industrial cameras, calibrated and placed in specific positions in order to completely survey an object within few seconds, instead of utilizing a digital camera mounted on a tripod and manually moved around the object. The LED number in the system will be increased in order to boost its flexibility and take advantage of light directionality for better surface inspection. For some particular objects, like a 3D inspection of plants, using LEDs with infrared light can also be useful. Spatially Varying BRDF will be also investigated for a better surface rendering.

## Figures and Tables

**Table 1:** Objects and acquisitions specifications.

Object	Size (mm)	f/Stop	Exposure Time (s)	Focal Length (mm)	GSD (mm)
A	240 × 150	1/16	1/8	60	0.02
B	160 × 200 × 30	1/16	1/8	60	0.02
C	140 × 50 × 40	1/22	1/4	60	0.02
D	50 × 50 × 40	1/22	1/8	60	0.02
E	25.75 × 25.75 × 2.2	1/22	1/8	105	0.01
F	100 × 100 × 10	1/22	1/30	60	0.02

**Table 2:** The estimated residuals of the ridge height (µm) between the proposed method and reference data from point 1 to point 13.

	P1-P2	P2-P3	P3-P4	P4-P5	P5-P6	P6-P7	P7-P8	P8-P9	P9-P10	P10-P11	P11-P12	P12-P13
Reference	53.79	60	54.03	59.73	58.53	58.03	56.03	58.63	57.7	51.8	52.85	52.05
Proposed	55.683	57.523	53.52	60.4	54.46	54.63	53.11	62.07	65.59	51.35	41.55	46.61
Residual	1.893	−2.477	−0.51	0.67	−4.07	−3.4	−2.92	3.44	7.89	−0.45	−11.3	−5.44

**Table 3:** The estimated residuals of the ridge height (µm) between the proposed method and reference data from point 13 to point 23.

	P13-P14	P14-P15	P15-P16	P16-P17	P17-P18	P18-P19	P19-P20	P20-P21	P21-P22	P22-P23
Reference	55.56	56.56	56.8	52.77	46.97	47.7	49.8	49.4	55	63.4
Proposed	50.44	41	58.95	61.57	41.24	52.39	55	44.68	41.99	48.72
Residual	−5.12	−15.36	2.15	8.8	−5.73	4.69	5.2	−4.72	−13.01	−14.68

**Table 4:** The results of high frequency evaluation (µm) for the proposed method.

Mean of Residuals	Maximum Residual	RMSE	MAE
−2.46	−15.36	1.5	5.48

**Table 5:** Comparison of the proposed method to three state-of-the-art approaches. The results are in millimeters. The blue values represent the best performance and the red values represent the lowest performance.

Object	Proposed (Method A)	Xiong et al. [83]	Quéau et al. [50]	Peng et al. [51]
D	0.54	1.24	1.3	1.1
E	0.025	0.22	0.14	0.13
F	0.16	2.6	1.7	0.71

## Data Availability

Upon a reasonable request from the corresponding author.
